# Genomic divergence and a lack of recent introgression between commercial and wild bumblebees (*Bombus terrestris*)

**DOI:** 10.1111/eva.13346

**Published:** 2022-03-14

**Authors:** Cecilia Kardum Hjort, Josephine R. Paris, Peter Olsson, Lina Herbertsson, Joachim R. de Miranda, Rachael Y. Dudaniec, Henrik G. Smith

**Affiliations:** ^1^ 5193 Department of Biology Lund University Lund Sweden; ^2^ School of Natural Sciences Macquarie University Sydney Australia; ^3^ 3286 Biosciences College of Life and Environmental Science University of Exeter Exeter UK; ^4^ 5193 Centre for Environmental and Climate Science Lund University Lund Sweden; ^5^ 8095 Department of Ecology Swedish University of Agricultural Sciences Uppsala Sweden

**Keywords:** *Bombus terrestris*, bumblebees, introgression, pollination, selection, single nucleotide polymorphisms, structural variants, whole‐genome sequencing

## Abstract

The global movement of bees for agricultural pollination services can affect local pollinator populations via hybridization. When commercial bumblebees are of the same species but of different geographic origin, intraspecific hybridization may result in beneficial integration of new genetic variation, or alternatively may disrupt locally adapted gene complexes. However, neither the existence nor the extent of genomic introgression and evolutionary divergence between wild and commercial bumblebees is fully understood. We obtained whole‐genome sequencing data from wild and commercial *Bombus terrestris* collected from sites in Southern Sweden with and without long‐term use of commercially imported *B*. *terrestris*. We search for evidence of introgression, dispersal and genome‐wide differentiation in a comparative genomic analysis of wild and commercial bumblebees. Commercial *B*. *terrestris* were found in natural environments near sites where commercial bumblebees were used, as well as drifting wild *B*. *terrestris* in commercial bumblebee colonies. However, we found no evidence for widespread, recent genomic introgression of commercial *B*. *terrestris* into local wild conspecific populations. We found that wild *B*. *terrestris* had significantly higher nucleotide diversity (Nei's pi, *π)*, while the number of segregating sites (Watterson's theta, *θw*) was higher in commercial *B*. *terrestris*. A highly divergent region on chromosome 11 was identified in commercial *B*. *terrestris* and found to be enriched with structural variants. The genes present in this region are involved in flight muscle contraction and structure and pathogen immune response, providing evidence for differing evolutionary processes operating in wild and commercial *B*. *terrestris*. We did not find evidence for recent introgression, suggesting that co‐occurring commercial *B*. *terrestris* have not disrupted evolutionary processes in wild *B*. *terrestris* populations.

## 1 INTRODUCTION

Deliberate or inadvertent human‐assisted movement of non‐native species into new locations creates opportunities for novel ecological interactions (Bartz & Kowarik, 2019; Crispo et al., 2011; Keller et al., 2011). However, the movement of a species to a new region where a closely related species co‐occurs may also result in genetic exchanges and altered evolutionary outcomes (Crispo et al., 2011; Kanbe et al., 2008; Yoon et al., 2009, 2011). Genomic introgression, the exchange of genetic material between species or subspecies through hybridization and repeated backcrossing (McFarlane & Pemberton, 2019), can be either beneficial or detrimental to local populations. Beneficial effects include ‘genetic rescue’ whereby novel genetic variation is introduced into genetically depauperate populations, or through ‘adaptive introgression’ by creating novel selection pathways that may reduce extinction risk (McFarlane & Pemberton, 2019) or simply a general increase in genetic diversity to better adapt to changing environments (Nelson et al., 2017; Suni et al., 2017). Alternatively, detrimental effects include reduced genetic diversity and disruption of local co‐adapted gene complexes, with novel alleles replacing locally adapted ones (Roesti, 2018; Schumer et al., 2018). Such processes may lead to population declines and even local extinctions (Jensen et al., 2005; Keller et al., 2014; Todesco et al., 2016). Introgression is therefore recognized as a process of significance for wildlife conservation (Laikre et al., 2010; McFarlane & Pemberton, 2019; Todesco et al., 2016).

There are many historical examples where exotic species have been deliberately or accidentally introduced beyond their natural range, leading to hybridization or introgression between closely related species (Biedrzycka et al., 2012; Chazara et al., 2010; Escalante et al., 2014). The use of exotic bee species for crop pollination and honey production has played a major role in the global movement of different bee species, repeatedly creating conditions for hybridization and introgression among native and non‐native bees (Byatt et al., 2015; Goulson, 2010; Kraus et al., 2007), which in some cases, has resulted in inviable hybrids between species (Kanbe et al., 2008; Tsuchida et al., 2010). More recently, attention has turned toward the ecological and evolutionary consequences of commercially produced bumblebees for native pollinator health and genetic diversity, due to the industrial‐scale global transport and use of commercial bumblebees for agricultural crop pollination (Dafni et al., 2010; Goulson, 2010; Velthuis & Van Doorn, 2006).

Bumblebees have several characteristics that make them highly effective agricultural pollinators, such as varied tongue lengths, tolerance to a wide range of weather conditions, and large hairy bodies providing ample surface area for pollen attachment. In particular, their ability to buzz‐pollinate by shaking floral anthers with high frequency to release pollen (Nayak et al., 2020; Nielsen et al., 2017; Wahengbam et al., 2019) makes bumblebees very effective pollinators for some crops (e.g., tomatoes: Cooley & Vallejo‐Marín, 2021) and therefore difficult to replace in agricultural settings. Commercial and introduced bumblebees, however, may be detrimental to local wild pollinators. For example, they are known to compete for resources with local native bees (Hingston & McQuillan, 1998; Ings et al., 2005, 2006; Morales et al., 2013) and spread pathogens (Dafni et al., 2010; Evans, 2017; Meeus et al., 2011) (but see Trillo et al., 2021), thereby disrupting native plant–pollinator relationships (Aizen et al., 2019; Hingston & McQuillan, 1998). However, the implications of commercial and introduced bumblebees for the genetic integrity and evolutionary trajectory of native pollinators is little understood (Seabra et al., 2019).

Since the commercialization of bumblebees in the late 1980s for the purpose of tomato pollination, the industry has grown rapidly (Owen, 2018). By 2006, it was estimated that more than one million reared colonies had been transported for introduction across the world (Velthuis & Van Doorn, 2006). While the subspecies *B*.* terrestris terrestris* was initially used for domestication (Velthuis & Van Doorn, 2006), several of the other nine *B*. *terrestris* subspecies have since been domesticated (Rasmont et al., 2008) and introduced outside of their native ranges (Inari et al., 2005; Kraus et al., 2011; Schmid‐Hempel et al., 2014). These nine subspecies are assumed to be adapted to the environmental conditions of their native geographic ranges and differ in phenology, colour patterning, behavioural traits and parasite resistance (Rasmont et al., 2008). Since only a few countries (e.g., Canada, United States and Japan) have implemented trade regulations for the importation of commercial *B*. *terrestris* (or for certain subspecies; Lecocq et al., 2016; Moreira et al., 2015; Velthuis & Van Doorn, 2006), introgressive hybridization among nonlocal or non‐native species or subspecies poses a threat to the genetic integrity of native or local bumblebee populations (Ings et al., 2006; Kanbe et al., 2008; Tsuchida et al., 2010; Yoon et al., 2009, 2011). Several studies have found evidence of introgression between native and commercial subspecies of bumblebees in the Iberian Peninsula, and Western and Eastern Europe (Bartomeus et al., 2020; Cejas et al., 2018, 2020; Moreira et al., 2015; Seabra et al., 2019). In other cases, introgression between commercial and native subspecies of bumblebees has not been detected, such as in the UK (Hart et al., 2021) and New England, USA (Suni et al., 2017).

Genetic differentiation between wild and commercial bumblebees is of further importance to investigate since different selection pressures may arise from the process of domesticating commercial bumblebees. Although there are no artificial selective breeding programs specifically for bumblebees (Lecocq, 2019), and genetic divergence between commercial and wild bumblebees is not necessarily expected, only the fact that they are raised in an artificial environment may result in divergence. Accordingly, studies have found significant pairwise genetic differentiation (*F*_ST_) between commercial and wild‐caught *Bombus impatiens* and *B*. *terrestris* within New England, USA, and within the Iberian Peninsula (Seabra et al., 2019; Suni et al., 2017). Although the drivers of this differentiation have not been thoroughly investigated, this suggests that demographic processes and natural selection may be operating differently on commercial and wild bumblebees. Thus, the identification of introgression among differentially selected bumblebees could result in novel alleles replacing locally adapted ones, interfering with processes of local adaptation. To our knowledge, no studies have investigated whether there is introgression between commercial and wild *B*. *terrestris* in Northern Europe. Additionally, to date, studies investigating introgression in bumblebees have used either microsatellites or reduced representation approaches (i.e., Restriction‐site Associated DNA sequencing—‘RADseq’ or Genotype By Sequencing—‘GBS’) and have therefore been limited in their ability to detect introgressive hybridization and its role in selection processes in wild and commercial bumblebees (Bartomeus et al., 2020; Cejas et al., 2018, 2020; Moreira et al., 2015; Seabra et al., 2019; Suni et al., 2017). Here, we use whole‐genome sequencing (WGS) data, which represents an opportunity to examine introgression, selection and evolutionary divergence at high resolution, using high‐density genetic markers across the entire genome, from commercial (most likely subspecies *B*. *t*. *terrestris* and/or *B*. *t*. *dalmatinus*; Goulson, 2010) and wild‐caught *B*. *terrestris* (dominant subspecies *B*.*t*. *terrestris*; Rasmont et al., 2008) sampled across the southernmost region of Sweden. This area is dominated by agriculture and includes a widespread but localized long‐standing use of commercial bumblebees for pollination, for example fruit, berries and tomatoes. We ask: (1) Is there evidence for genomic introgression between wild and commercial populations of *B*. *terrestris*? And (2) do genome‐wide selection signatures differ between commercial and native bumblebees? Our study has implications for commercial practices involving the distribution and containment of bumblebees, understanding adaptive genetic variation in commercial and native bumblebees and, therefore, monitoring the evolutionary resilience of pollinators in a rapidly changing world.

## 2 MATERIALS AND METHODS

### 2.1 Experimental design

Ideally, introgression should be investigated before and after the introduction of commercial bumblebees in a landscape, which however is logistically infeasible as detectable signals are only expected to develop over time. Instead, we used an experimental landscape design, which assumes that contemporary spatial ecological patterns are roughly equivalent to changes over time (cf. Pickett, 1989), to determine the occurrence and extent of genomic introgression of commercial *B*. *terrestris* genetic material into wild *B*. *terrestris* genomes. This was done by comparing the genomes of commercial *B*. *terrestris* with those of (wild) *B*. *terrestris* caught at sites with long‐term use of commercial *B*. *terrestris* used for pollination services (hereafter ‘experimental sites’) with those of wild *B*. *terrestris* caught at sites with no history of commercial bumblebee use (hereafter ‘control sites’). Control sites were at least 1500 m from the nearest commercial bumblebee colony. Commercial *B*. *terrestris* were collected at the experimental sites and sites located in our general study area. To account for potential environmental variability between the experimental and control sites, we chose control sites that were located in areas with similar high agricultural land cover as the experimental sites. All sampling sites were located on the same latitude, and hence, temperature and precipitation did not vary markedly between sites (Table S1; data from WorldClim v2.1, Fick & Hijmans, 2017).

### 2.2 Sampling collection and study area

#### 2.2.1 Wild‐caught bumblebees

**FIGURE 1:**
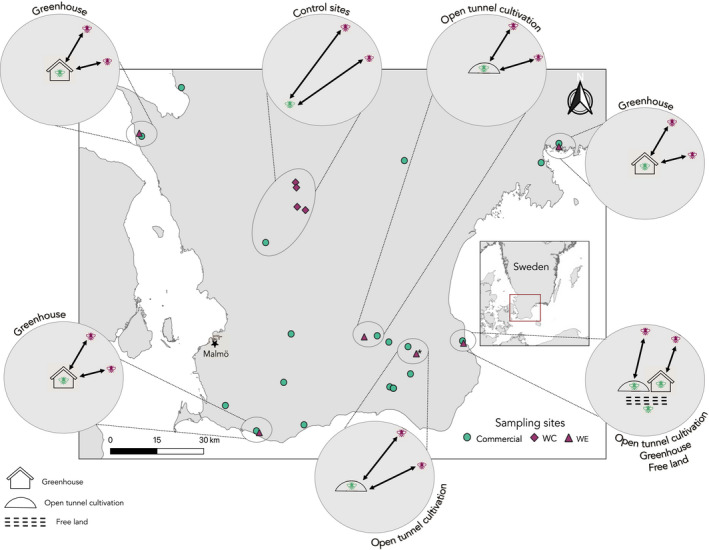
Sampling locations for all free‐flying wild collected bumblebees and locations of the commercial hives in Southern Sweden. Free‐flying female workers collected at experimental sites (WE) in 2018 and 2019, where commercial bumblebees have been used for 12–27 years are shown as dark purple triangles (site from 2019 has an ‘*’ next to the symbol) and were collected 700–3000 m from the closest location with commercial bumblebees. Free‐flying female workers collected at control sites (WC) at least 15 km from the nearest location with commercial bumblebees are shown as dark purple diamonds. The locations of the commercial hives (CB) are shown as green circles (commercial, *n* = 18). Six of the zoomed‐in circles illustrate the ‘experimental sites’ and the three different agricultural practices: greenhouse; open tunnel‐cultivation and free land, which use commercial bumblebees for pollination services. The final zoomed‐in circle illustrates the ‘control sites’

Seventy‐eight free‐flying ‘wild’ female diploid worker *B*. *terrestris* (WB) were collected across ten sites in Southern Sweden during July 2018 (nine sites) and July 2019 (one site) (Figure 1; Table 1). At five ‘experimental’ sites, commercially reared bumblebees had been used for 12–27 years (experimental sites) for pollination in either greenhouse cultivation of tomatoes (three sites), tunnel cultivation of berries (one site) or a combination of greenhouse (tomato), tunnel (berries) and free land (berries and apples) cultivation (one site), at a stocking rate of 50 to 350 colonies/year. At these experimental sites, wild free‐flying bumblebees (hereafter, called wild experimental ‘WE’) were collected between 700–1000 m from the closest greenhouse, tunnel and/or free land with commercial bumblebee colonies. At the sixth experimental site, commercially reared bumblebees have been used since 2013 (except during the years of 2015 and 2017) at a stocking rate of 12 colonies/year. At this site, wild free‐flying bumblebees were collected at 3000 m from the closest free land with commercial bumblebee colonies (hereafter, called wild experimental ‘WE’). The site was added later to increase the total sample size, but only three individual samples from this site were included in the dataset used for analyses due to quality control of the dataset and identification of full siblings. The selection of sampling locations for all experimental sites allows for the detection of introgression while minimizing the incidence of commercial ‘escapees’ since the mean foraging distance for *B*. *terrestris* workers are <300 m from the colony (Wolf & Moritz, 2008), although foraging trips may be longer than 700 m (Goulson, 2010).

**TABLE 1:** Sampling of *B. terrestris* in Southern Sweden

Site ID	Sample groups	CB colonies present	Cultivation type	# of CB colonies/year	# of years with CB colony usage	# of CBs collected	# of free‐flying WBs caught	Distance from nearest CB colony	Sampling/collection date
V1	WE	Yes	Greenhouse	300–350	25	–	7	1000 m	03–08–2018
V2	WE	Yes	Greenhouse	NA*	18	–	9	1000 m	02–08–2018
V3	WE	Yes	Greenhouse	300–350	23	–	9	900 m	03–08–2018
T2	WE	Yes	Tunnel	50	12	–	12	1000 m	30–07–2018
T6	WE	Yes	Greenhouse, tunnel, free land	25	27	–	14	700 m	30–07–2018
1326	WE	Yes	Free land	12	3	–	5	3000 m	17–07–2019
E1	WC	No	–	–	–	–	12	20 km	25–07–2018
E2	WC	No	–	–	–	–	6	19 km	25–07–2018
E3	WC	No	–	–	–	–	2	15 km	25–07–2018
E4	WC	No	–	–	–	–	1	15 km	25–07–2018
V1	CB	Yes	Greenhouse	300–350	25	1	–	–	27–07–2018
V2	CB	Yes	Greenhouse	NA*	18	1	–	–	27–07–2018
V3	CB	Yes	Greenhouse	300–350	23	1	–	–	27–07–2018
V5	CB	Yes	Greenhouse	3	NA	1	–	–	27–07–2018
V6	CB	Yes	Greenhouse	11	27	1			27–07–2018
T1	CB	Yes	Tunnel	6	NA	1	–	–	27–07–2018
T5	CB	Yes	Tunnel, free land	3	NA	1	–	–	27–07–2018
T6	CB	Yes	Greenhouse, tunnel, free land	25	27	1	–	–	27–07–2018
I66025	CB	Yes, freestanding	–	NA	NA	1	–	–	27–07–2018
I87032	CB	Yes, freestanding	–	NA	NA	1	–	–	27–07–2018
I40017	CB	Yes, freestanding	–	NA	NA	1	–	–	27–07–2018
132006	CB	Yes, freestanding	–	NA	NA	1	–	–	27–07–2018
I59019	CB	Yes, freestanding	–	NA	NA	1	–	–	27–07–2018
I16004	CB	Yes, freestanding	–	NA	NA	1	–	–	27–07–2018
I59020	CB	Yes, freestanding	–	NA	NA	1	–	–	27–07–2018
136014	CB	Yes, freestanding	–	NA	NA	1	–	–	27–07–2018
133007	CB	Yes, freestanding	–	NA	NA	1	–	–	27–07–2018
I59021	CB	Yes, freestanding	–	NA	NA	1	–	–	27–07–2018

Note

The name of the sampling sites and the name of the three sampling groups are illustrated in the table. Indication if commercial colonies are present in the agricultural practices located in our sampling sites, the cultivation type, the number of commercial bumblebee (CB) colonies used on a yearly basis, the number of years with CB colony usage for the agricultural practices used in the study, the number of free‐flying wild bumblebees (WBs) caught at each site and the distance in meters from each sampling site to the nearest CB colony is also illustrated in the table.

We collected the free‐flying and commercial bumblebees between 17/07/2018 and 03/08/2018.

Abbreviations: WE, wild experimental bumblebees (*n* = 57); CB, commercial bumblebees (*n* = 18) and WC, wild control bumblebees (*n* = 21). NA*, the exact number of CB colonies used per year is not known but the greenhouse tomato cultivation is of large scale and has been operating since 2000.

Free‐flying *B*. *terrestris* workers (hereafter, called wild control ‘WC’) were also collected at four ‘control sites’ at least 15–20 km from the nearest location of commercial bumblebee use (data collection summarized in Table 1). These sites were selected to maximize the distance between where wild ‘control’ *B*. *terrestris* were sampled, and the location of the closest commercial bumblebee colony(ies). Out of necessity, the sites were relatively close to each other since it proved difficult to find locations in Southern Sweden where we could safely assume that commercial bumblebees have not been used or are currently being used. We ensured the absence of use of commercial bees by avoiding known locations using bumblebees and by collecting customer postcodes of sales records from the major bumblebee importers to avoid these areas.

The locations of commercial bumblebees (experimental sites) were identified by contacting growers producing bumblebee‐dependent crops in greenhouses or tunnels; the names of enterprises and their exact location remain confidential by agreement. We collected all encountered foraging or flying wild bumblebees using sweep nets and brought back specimens to the laboratory in a cooling box, preserved in 70% ethanol and stored at −20°C.

#### 2.2.2 Commercial bumblebees

We collected 18 commercial bumblebees (hereafter ‘CB’) directly from 18 different colonies, one bee per colony (Biobest Group NV, Belgium) (Table 1). Four of these colonies came from the greenhouse, tunnel and free‐land cultivations where wild experimental (WE) samples were also collected. The remaining 14 colonies were from the same general study area as our wild experimental sites (WE) and at least 15 km from our wild control (WC) sampling sites (Figure 1). All commercial bumblebees originated from the same supplier. We preserved all bumblebees in 70% ethanol and stored them at −20°C.

### 2.3 DNA extraction, barcoding and sequencing

Genomic DNA was extracted from the head and two legs of each bumblebee using a Qiagen Blood & Tissue Extraction Kit (QIAGEN GmbH) following a modified version of the manufacturers Supplementary Protocol (Text S1). To confirm species identification of the free‐flying bumblebees, the COI mitochondrial gene was amplified in all samples according to Wahlberg and Wheat (2008) (Text S2). COI sequences were compared with both complete and partial mitochondrial genome sequences from *B*. *terrestris* in GenBank using Nucleotide BLAST to confirm that all samples were *B*. *terrestris*, using a 100% match rate as a threshold. DNA samples (1100–3000 ng DNA per sample) were prepared as sequencing libraries by the National Genomics Infrastructure (NGI) Sweden, SciLifeLab (Stockholm University) using an (in‐house) automated version of the Illumina protocol ‘TruSeq DNA PCR‐Free Sample prep’ (Part #15036187 Rev. D, June 2015) on an Agilent Bravo automated liquid handling platform. Sequencing was performed by SciLifeLab on an Illumina NovaSeq6000, in a single lane on an S4 flow cell, generating paired‐end reads of 2 × 150 bp in length with an average depth of coverage between 18–82× per sample (average depth of 33× across all samples).

### 2.4 Whole‐genome assembly and SNP variant calling

Raw reads were demultiplexed and converted to FASTQ using bcl2fastqv2.20.0.422 using the CASAVA software suite provided by the National Genomics Infrastructure (NGI) Sweden and Science for Life Laboratory (SciLifeLab) (Stockholm University). Standardized bioinformatic quality control checks were performed by SciLifeLab prior to data delivery (Table S2). Reads were mapped to the *B*. *terrestris* reference genome (Sadd et al., 2015) (assembly accession: GCF000214255.1) using the portable workflow for whole‐genome sequencing analysis in ‘Sarek’ (Garcia et al., 2020). Reads were mapped using Burrows‐Wheeler Aligner (BWA‐mem) v0.7.5 (Heng Li & Durbin, 2009) and resulting alignments were deduplicated using MarkDuplicates GATK v4.1.4.1 (Van der Auwera et al., 2013). Variants were called using two haplotype‐based variant detectors; HaplotypeCaller in GATK v4.1.4.1 (Poplin et al., 2017) and FreeBayes v1.3.1 (Garrison & Marth, 2012). An intersect of the variants of both methods were used for downstream analyses.

For GATK, SNPs called with HaplotypeCaller were evaluated and filtered for quality using GATK recommended standard parameters (Poplin et al., 2017). The resulting 5,518,620 SNPs were further filtered to retain only biallelic SNPs and a minimum and maximum mapping coverage of 10 and 300, respectively, using VCFtools v0.1.16 (Danecek et al., 2011) resulting in a final VCF file containing 5,359,624 SNPs. For Freebayes, SNPs were filtered for mapping quality of >30 and a minimum mapping coverage of 10, using the VCFfilter tool available in Vcflib (https://github.com/vcflib/vcflib). A VCF file containing 3,500,520 SNPs was generated. The two filtered VCF files (generated by GATK and FreeBayes) were intersected to create a confidence set of known SNPs using BCFtools v.1.10 (http://samtools.github.io/bcftools/).

Final filtering of the VCF file consisted of retaining SNPs that had been called in 50% of individuals from each of the three sampling groups at a minor allele frequency (MAF) of 3% across the whole dataset. Individuals with more than 70% missing sequencing coverage were removed from the dataset using VCFtools v.0.1.16 (Danecek et al., 2011). We used the relatedness2 option in VCFtools v.0.1.16 (Danecek et al., 2011), based on the methods of Manichaikul et al. (2010), to identify and remove full siblings from the dataset. For analyses sensitive to linkage, we used an additional VCF file, which was filtered using indep‐pairwise in Plink v.1.90b4.9 (parameters of 50, 5 and 0.2) (Purcell et al., 2007). Analysis was performed on the final set of 652,002 SNPs from the 18 assembled *B*. *terrestris* chromosomes (excluding scaffold sequences).

### 2.5 Genetic structure and introgression

**FIGURE 2:**
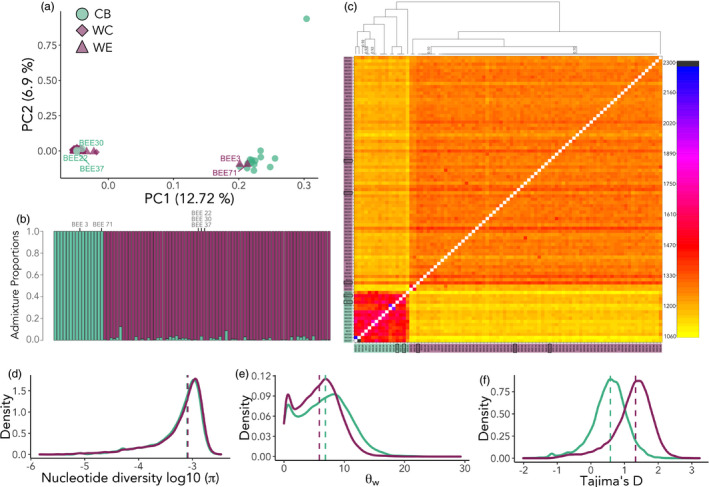
Population structure and genetic diversity of *B*. *terrestris* individuals (*n* = 89). (a) principal components analysis (PCA) where two primary clusters are defined; the commercial bees (CB) group forms its own cluster, and the wild experimental (WE) and wild control (WC) groups cluster together (wild bees—‘WB’). Two WB individuals cluster with the CB group (i.e., ‘escapees’). Three CB individuals cluster with the WB group (i.e., ‘drifters’). (b) Genetic admixture for each individual sample shows *K* = 2. Cluster 1 (green, CB) comprises commercial bees and cluster 2 (purple, WB) comprises wild individuals. Individuals considered to be escapes and drifters are marked on the plot. (c) Simple co‐ancestry matrix visualized as a heatmap generated by fineSTRUCTURE and CHROMOPAINTER. The colour of each cell in the matrix shows the number of expected shared genetic chunks copied from a donor genome (column) to a recipient genome (row). Individuals considered to be escapes and drifters are marked on the plot with boxes around the sample name. Support for the branches on the co‐ancestry tree are 1, unless stated otherwise on the plot. (d) Nucleotide diversity (π) on a log10 scale; (e) Watterson's theta (θw); (f) Tajima's *D* for the CB (green) group and WB (purple) group. Dashed lines represent medians

We first performed a principal component analysis (PCA) on the whole dataset using the linkage‐pruned SNP dataset. Resulting eigenvectors were plotted in R using ggplot2 (Wickham, 2016). We also performed two separate PCAs, one on the CB group and one on the WE and WC together (hereafter wild bumblebees, ‘WB’) to identify any further genetic sub‐structuring within each of the two groups. Before grouping the WE and WC groups together, we calculated pairwise *F*_ST_ (Weir & Cockerham, 1984) between the two groups using PopGenome v.2.7.5 (Pfeifer et al., 2014). We performed two separate genome‐wide individual ancestry analyses to further characterize genetic structure and identify potentially introgressed individuals using ADMIXTURE v.1.3.0 (Alexander & Lange, 2011) and fineSTRUCTURE v.4.1.1 (Lawson et al., 2012). For ADMIXTURE, in order to choose the appropriate value of *K* (i.e., the optimal number of clusters within the dataset), we ran a cross‐validation procedure with *K* values of 1–5 and with a 10‐fold cross‐validation (CV). We performed 10 separate runs of the algorithm, using a different random seed for each run, taking the average of the 10 runs for our final result (Figure S3). The same cross‐validation procedure was run separately for the CB group and the WB group to identify any further sub‐structure within groups. The CV error rates are reported as an output logfile for each value of *K* (Figure S3). For fineSTRUCTURE, we first phased the SNP‐dataset using BEAGLE v.3.1.2 (Browning & Browning, 2009) and then SHAPEIT v2.r904 (Delaneau et al., 2012) to create a recombination map in CHROMOPAINTER v.0.0.4 (Lawson et al., 2012). The recombination map was implemented in fineSTRUCTURE to generate a co‐ancestry matrix (Figure 2b); fineSTRUCTURE was run with the following parameters: 10 minimum EM iterations (used for chromopainter), default of 10000 minimum number of SNPs for EM estimation and 200000 total MCMC iterations.

### 2.6 Genetic diversity

We used Hierfstat v.0.5.8 (Goudet, 2005) to calculate the inbreeding coefficients (*F*_IS_) for the wild and commercial bumblebee groups. Then, to explore genome‐wide diversity within each of these groups, we calculated the number of segregating sites (Watterson's theta, θw) (Watterson, 1975), nucleotide diversity (Nei's pi, π) (Nei, 1979) and Tajima's D as a neutrality test of any deviations between θw and π (Tajima, 1989) in 10kb windows genome wide and per chromosome using PopGenome v.2.7.5 (Pfeifer et al., 2014). Upper (95%) and lower (5%) CIs were calculated for all statistics using the R package stats v.3.6.2 (R Core Team, 2018). Statistical significance of *F*_IS_, θw, π and Tajima's D between the two groups was evaluated using a Wilcoxon test with the R package rstatix v.0.6.0 (Kassambara, 2020).

### 2.7 Selection detection in commercial and wild bumblebees

To explore differentiation along the genome between wild and commercial bumblebees, we calculated pairwise *F*_ST_ (Weir & Cockerham, 1984) and mean *F*_ST_ per chromosome in 10 kb nonoverlapping windows using PopGenome v.2.7.5 (Pfeifer et al., 2014). As *F*_ST_ is only a relative measure of divergence and is strongly influenced by the level of within‐population diversity, we also used the cross‐population extended haplotype homozygosity statistical test (XP‐EHH) in selscan v1.2.0 (Szpiech & Hernandez, 2014). The method is used to detect recent directional selection via selective sweeps and compares haplotype lengths between two populations (Sabeti et al., 2007). To identify stretches of extended homozygotic haplotypes in each group (CB and WB), we computed XP‐EHH scores using the other group as the diversity reference. XP‐EHH scores were standardized across 10 kb windows. To identify regions of the genome potentially under selection, we identified regions in which 10 kb windows overlapped in high *F*_ST_ (10 kb windows above the 99% CI) and XP‐EHH scores. Since selection and genomic rearrangements can cause shifts in localized heterogeneity along chromosomes, we then used lostuct v.0.9 (Han Li & Ralph, 2019) to search for patterns of local changes in ancestry by calculating local PCAs across chromosomes in 100bp windows. We visualized local chromosomal deviations in population structure using multidimensional scaling plots (MDS).

### 2.8 Structural variant detection

To detect structural variants (SV) from our short‐read whole‐genome sequencing data, we used two SV detection programs: smoove v.0.2.5, which uses lumpy (Layer et al., 2014) and Breakdancer v.1.4.5 (Fan et al., 2014). Both SV callers were run for the CB and WB groups separately. For smoove outputs, we did not consider SVs that were marked as ‘imprecise’, <=1kb in length, and which had read pair support lower than the median for each group (CB: 39; WB:35). For Breakdancer outputs, we removed SVs that had a quality score <99, <=1kb in length, and which had a fewer number of reads than the median for each group (CB: 50, WB: 334).

### 2.9 Assessment of intrachromosomal linkage disequilibrium

To assess patterns of intrachromosomal linkage, we calculated linkage disequilibrium (LD) between all pairs of SNPs (reported as squared correlations) for both the CB and WB groups together and also separately using Plink v.1.90b4.9 (Purcell et al., 2007). SNPs were filtered for minor allele frequency (MAF) of 0.1 and thinned at 5kb using vcftools v.0.1.16 (Danecek et al., 2011). Pairwise LD was visualized using the R package LDheatmap v.1.0–4 (Shin et al., 2006).

### 2.10 Gene annotation

To explore candidate genes within identified regions of high divergence between wild and commercial bumblebees, we extracted the gene ID, start and end position, accession IDs and gene name identified from the *B*. *terrestris* reference genome. Identified genes were used in gene ontology enrichment analysis (GO) using the R package *biomaRt* (Durinck et al., 2009) via Bioconductor v. 3.12 (Yu, 2014). The reference list of genes was also matched with the KEGG (Kyoto Encyclopaedia of Genes and Genomes) ENZYME database to identify potential enzymes and their function using *biomaRt*.

## 3 RESULTS

### 3.1 Data processing and quality control

We obtained a total of 5,672,780,248 reads across all genotyped individuals and the mean number of reads per individual was 5,909,1461 (Table S3). Of the total 96 sequenced individuals, three had >70% missing data and were therefore removed from the dataset. We identified five wild‐caught full siblings (one sibling pair and one sibling trio). Based on the estimated kinship coefficient (1^st^ degree sibling: 0.177– 0.354; Manichaikul et al., 2010), we retained two unrelated individuals of the five in the dataset. We also identified one additional individual with a highly negative estimated kinship coefficient of −0.9 in all pairwise tests, indicative of much lower relatedness than expected by chance, and increased genetic divergence (Manichaikul et al., 2010). We suspect that this individual was erroneously identified as our target species during COI barcoding and it was therefore removed. The final dataset consisted of 89 individuals for analysis.

### 3.2 Genetic structure of commercial and wild bumblebees

The PCA showed two defined clusters, where the wild bumblebees (WB) cluster together and the commercial bees (CB) formed a separate cluster along PC1 (12.7% of the variance) (Figure 2a). There was no further genetic substructure observed within either the CB or the WB group when these were analysed separately using PCA (Figures S1 and S2). Pairwise *F*_ST_ between the WE and WC groups was low (*F*_ST_ = 0.0004), and we therefore grouped the WE and WC groups together in all following analyses. One CB individual (Sample 25) showed genetic distinctiveness along PC2 (6.9% variance). We did not find evidence that the underlying data for this individual were biased in any way, that is in the number of reads, coverage, alignment rate, or missing data. We did however find that this individual had considerably higher observed homozygosity (Ho: 0.92; He: 0.68) and *F*_IS_ (0.980), suggesting that the low genetic diversity of this sample skewed its placement in PCA space. The PCA showed no clear evidence for shared genetic assignment between the wild and commercial bees. Notably, two of the WE samples (samples 3 and 71) from two different experimental sites (V2 and T6, see Table 1) clustered with the CB group. We believe that these individuals represent commercial ‘escapees’ that were caught foraging outside of the cultivation (greenhouse, open tunnel cultivations and free land). They also grouped within the CB cluster (Figure 2b,c). Additionally, three of the CB samples (samples 22, 30 and 37) were genetically assigned to the WB group (Figure 2b,c), which is most likely due to wild bees entering the commercial bumblebee nest, either accidentally due to disorientation (‘drifting’) or deliberately, for theft of resources or shelter, which is a documented phenomenon among both wild and commercial bumblebees where bees visit non‐natal colonies (Zanette et al., 2014). We did not find any evidence for genetic admixture in the identified drifters or escapees.

The cross‐validation procedure of ADMIXTURE to identify potentially introgressed individuals performed on the two groups (CB and WB) gave the highest support for one genetic cluster, *K* = 1 (0.488) (Figure S3, see Figure S4 for variation across runs). However, two genetic clusters, *K* = 2, also exhibited a low cross‐validation error rate (0.501). Arguably, *K* = 1 and *K* = 2 often cannot be distinguished and it is recommended to explore multiple *K* values of the dataset (Janes et al., 2017) (see Figure S5 for *K* = 3). Apparent from the *K* = 2 result (and *K* = 3, see Figure S5) was a lack of admixed samples (Figure 2b), since none of the WB samples had high ancestry proportions derived from the CB group (see Figure S6 for bootstrapped admixture proportions). Again, this was also evident for the drifters and escapees. The cross‐validation procedure run with the CB group and the WB group separately exhibited the highest support for *K* = 1 for both groups (CB = 0.64, WB = 0.48) (Figure S3).

The fineSTRUCTURE coancestry matrix showed a clear separation between the CB and the WB groups, with members of the former tending to share the highest ancestry with each other (Figure 2c). Some genetic substructuring was evident within the two groups. Sample CB25 (also observed as an outlier in the PCA, Figure 2a) shared the lowest ancestry with the majority of the other CB individuals, as well as with all individuals in the WB group. Samples 3 and 71 from the WB group were clearly assigned as commercial escapees (Figure 2a), as further demonstrated by ADMIXTURE (Figure 2c). Assessment of relatedness from vcftools showed that there were 2nd and 3rd degree relatives present in the dataset. Two pairs of CB individuals were 2nd degree relatives (sample 27 and 40, kinship coefficient of 0.101; sample 24 and 38, kinship coefficient of 0.089) and one pair of CB individuals were 3rd degree relatives (sample 28 and 31, kinship coefficient of 0.041). Two of the WB samples (35 and 78) were identified as 2nd degree relatives (kinship coefficient of 0.100), sharing higher relatedness with each other compared to all other WB samples, and as a result were the least related to the CB group among the WB samples (also evident in Figure 2c). There were no ancestry patterns indicative of admixture between the two main groups (Figure 2c), which again indicates a lack of introgression. This was also true for the drifters and escapees, which also did not show any evidence of high co‐ancestry between each other, nor with individuals in their genetic group, again supporting that they are not admixed individuals. The clustering dendrogram formed 13 lineages, where nine of the lineages consisted of the CB samples and four of the lineages consisted of the WB samples (top of Figure 2c).

### 3.3 Genetic diversity

All our quantified diversity statistics (Watterson's theta (θw), Nei's pi (π), Tajima's *D* and *F*_IS_) were statistically different between the WB and CB groups. Although genome‐wide π distributions were similar (Figure 2d), the WB group had a higher median π (0.00082, 95% CIs: 0.000–0.0016) compared to the CB group (0.00077, 95% CIs: 0.000–0.0016) (*p* < 0.0001). Higher median π in the WB group was also reflected in the per‐chromosome calculations (Table S4). In contrast, the median θw was significantly lower in the WB group (5.85, 95% CIs: 0.549–10.999) compared to the CB group (6.86, 95% CIs: 0.527–13.272) (*p* < 0.0001), also apparent from the genome‐wide distribution of the statistic (Figure 2e). The number of segregating sites (θw) per chromosome was also marginally higher in the CB group (Table S4). The discrepancy between π and θw was reflected in the Tajima's D values. Tajima's D in the CB group was 0.56 (95% CIs: −0.390 to 1.310) and in the WB group 1.33 (95% CIs: 0.107–2.009) (*p* < 0.001). As a result, the WB showed a right‐skewed distribution of positive Tajima's *D* (Figure 2f), indicative of a deficit (*D* > 0) of rare alleles. Global *F*_IS_ values within the CB and WB group were generally low, 0.044 and −0.009, respectively, and both included 0 in their 95% CIs (CB: −0.294 to 0.576; WB: −0.175 to 0.206). Although the *F*_IS_ values did not show significant inbreeding within either of the two groups, there was significant difference in *F*_IS_ between the CB and WB groups (*p* < 0.001).

### 3.4 Genome‐wide differentiation

**FIGURE 3:**
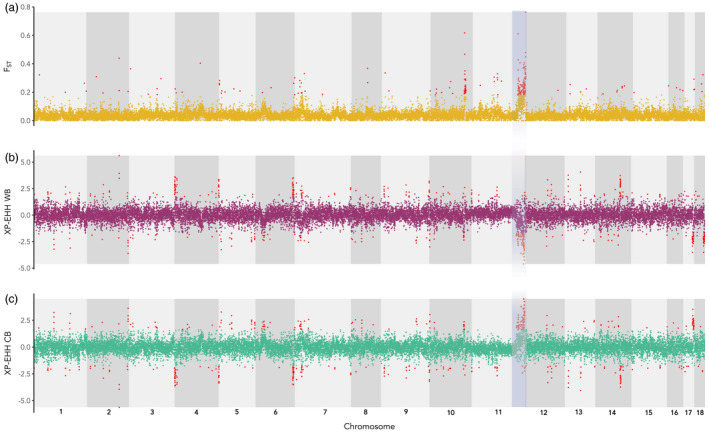
Genome scans run across sliding windows of 10 kb, where the blue highlights the region on chromosome 11 containing SNPs with increased differentiation that are under putative selection; red SNPs indicate the upper 99% and lower 1% confidence intervals. (a) The genetic differentiation (pairwise *F*_st_) between the two groups; (b) standard mean XP‐EHH scores for the WB group; (c) standard mean XP‐EHH scores for the CB group

**FIGURE 4:**
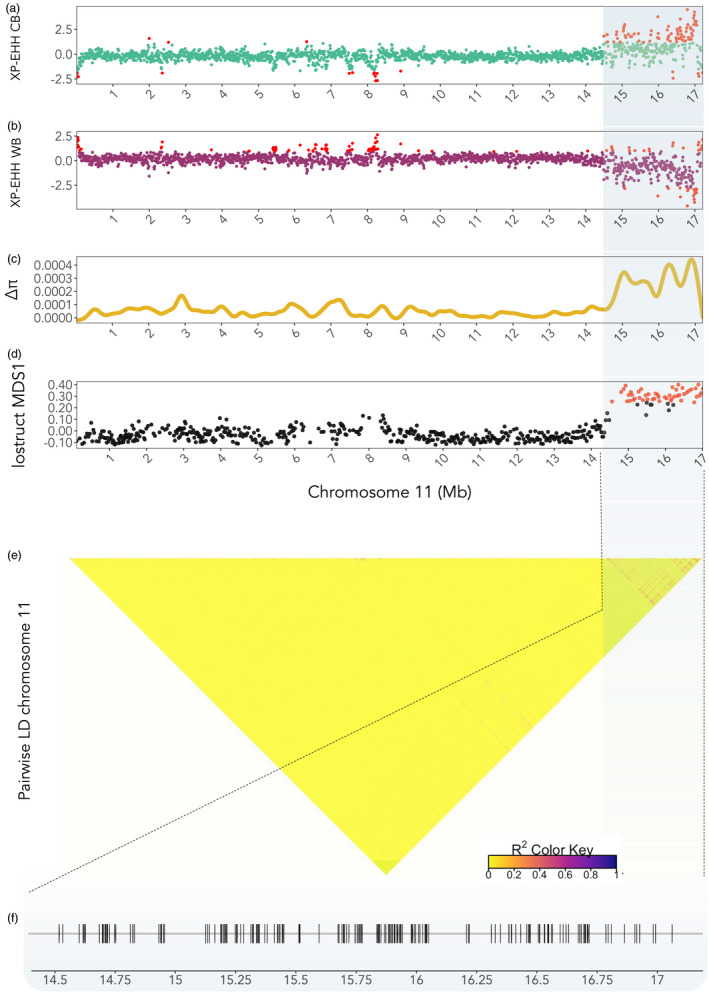
(a) standard mean XP‐EHH scores for chromosome 11 for CB, and (b) for WB; (c) Delta nucleotide diversity along chromosome 11; red SNPs indicate the upper 99% and lower 1% confidence intervals; (d) Multidimensional scaling (MDS) plot of chromosome 11. Each point represents a window where the red points show windows with increased genetic differentiation from the rest of the chromosome; (e) pairwise linkage disequilibrium (LD) heatmap, calculated using r squared. Blue highlights the region containing SNPs with increased differentiation, or the SNPs in high LD (e); (f) annotated genes (*n* = 178) for outlier SNPs on chromosome 11

The median global *F*_ST_ across the whole genome was moderate to low between the CB and WB groups, 0.034 (99% CIs: 0–0.182). When analysing individual chromosomes, the median *F*_ST_ ranged from 0.028–0.041 (Table S5). When we visualized *F*_ST_ across the whole genome, chromosomes 10 and 11 both had notable peaks of high *F*_ST_ (Figure 3a). Compared to the rest of the genome, chromosome 11 also showed positive XP‐EHH in the CB group, and respectively negative XP‐EHH in the WB group (Figure 3b and 3c). Across the genome, chromosome 11 showed the highest genetic differentiation between the CB and WB (median *F*_ST_ = 0.041) with a clear peak of high *F*_ST_ values at the tail‐end region of chromosome 11 (2500 Mb long: position 14,500–17,000 Mb) (Figure 4a). Using the XP‐EHH statistic with the WB group as a reference, we identified a region of highly positive XP‐EHH values at the tail‐end of chromosome 11, indicating an excess of long stretches of haplotype homozygosity, indicative of potential selection in the CB group (Figure 4a, excess of long haplotype homozygotes also evident in Figure S7 dendrogram). The same region showed highly negative XP‐EHH values when running the CB group as a reference (Figure 4b). Additionally, the difference in nucleotide diversity (*Δ π*) between the two groups was evident in the same region (Figure 4c), as well as chromosomal deviations as identified by *lostruct* in this region in comparison to the rest of chromosome 11 (Figure 4d). When the CB and WB group were run together, SNPs in this region also showed evidence of increased linkage in the CB group (Figure 4e). No strong linkage between SNPs was observed within the CB and WB group in the region when run separately (Figures S8 and S9). The short‐read tests performed to uncover potential SVs in the candidate region showed an enrichment of SVs in general, but we did not detect a large structural variant encompassing the whole candidate region (Tables S6–S9). Enriched SVs included inversions, deletions, translocations and duplications.

Chromosome 10 also showed a clear peak of high *F*_ST_ values indicating genetic differentiation between the CB and WB groups (median *F*_ST_ = 0.037) (Figure 3a). However, this region was much narrower than the region identified on chromosome 11 (260Mb, position: 11030–11290 Mb), and there was no clear evidence of either highly positive or negative XP‐EHH values in this region (Figure S10).

### 3.5 Gene functions under potential selection on chromosome 11

The 177 genes present in the candidate region on chromosome 11 (Figure 4f) were associated with 102 unique gene ontology (GO) terms (Table S10). Five GO terms were significantly enriched at *p* < 0.05, although these were nonsignificant after correction for the false discovery rate using multiple comparisons: calcium ion binding (*p* = 0.013), transcription initiation from RNA polymerase II promoter (*p* = 0.016), chitin binding (*p* = 0.025), DNA‐templated transcription, initiation (*p* = 0.043) and nucleic acid binding (*p* = 0.044). Two KEGG ENZYME classes were identified from the 177 genes: transferases and hydrolases (EC 2.7.4.3 in maps 00230 and 00730 and EC 3.2.1.14 in map 00520).

## 4 DISCUSSION

Using a whole‐genome sequencing approach, we investigated evidence for introgression and genomic divergence between commercial and wild‐caught *B*. *terrestris* in Southern Sweden. Despite the discovery of escaped commercial bumblebees among the wild‐caught group, as well as wild *B*. *terrestris* ‘drifters’ inside the commercial bumblebee colonies, we did not find evidence for recent genomic introgression among our samples. Although wild bumblebees had significantly higher nucleotide diversity (*π)* than commercial ones, the number of segregating sites (*θw)* was higher in commercial bumblebees. This discrepancy was supported by positive Tajima's *D* values in wild bumblebees. Selection scans across the genome revealed a highly divergent region on chromosome 11 in commercial bumblebees, as well as a high degree of linkage and chromosomal deviations within this region. This is indicative of differential selection processes operating in this region. Although we found no clear evidence for a single large structural variant (SV) associated with this divergent region, we found an overall enrichment of shorter SVs associated with this region. The divergent region contained genes involved in cellular processes that have previously been associated with flight muscle contraction and structure in insects (Bullard & Pastore, 2011; Cao & Jin, 2020; Kržič et al., 2010; Rusu et al., 2017), associated with pathogen immune response (Ramsey et al., 2017). The lack of introgression indicates that the extensive and historical use of commercial bumblebees in Southern Sweden has so far not affected the evolutionary processes within wild populations of *B*. *terrestris* via introgression.

### 4.1 Genetic structure and a lack of introgression

We identified two distinct genetic groups that separated wild bumblebees and commercial bumblebees. The groups showed no substantial evidence for shared recent genetic ancestry, indicating a distinct lack of introgression within Southern Sweden. We found subtle variation in genetic co‐ancestry among individuals within the two clusters, which was likely due to the presence of 2^nd^ and 3^rd^ degree siblings (Figure 2c). The dendrograms for each group (Figure 2c) showed that the commercial bumblebees consisted of nine lineages and the wild bumblebees had four lineages. This may reflect the use of *B*. *terrestris* queens originating from multiple locations in commercial breeding programs (Velthuis & Van Doorn, 2006). Notably, there was no genetic structure detected among the wild‐caught bumblebees, including bees sampled from both experimental (WE) and control sites (WC), which may be explained by the capacity of bumblebees to disperse distances of several kilometres (Kraus et al., 2009). The fact that we did not find any genetic structuring is consistent with previous studies which have evidenced (even at large spatial scales) low to moderate genetic differentiation among wild *B*. *terrestris* populations in mainland Europe (Estoup et al., 1996; Seabra et al., 2019; Silva et al., 2020) and wild *B*. *impatiens* populations in New England, USA (Suni et al., 2017).

That we did not find any introgression is concordant with some previous studies. Suni et al. (2017) did not detect any evidence of introgression in wild *B*. *impatiens* in New England, regardless of previous, current or no contact with commercial *B*. *impatiens*, whereas Hart et al. (2021) did not detect any introgression between wild *B*. *t*. *audax* and commercial *B*. *t*. *audax* and *B*. *t*. *dalmatinus* in the UK. In contrast, several studies in the Iberian Peninsula have shown clear evidence for introgression between local and commercial *B*. *terrestris* subspecies (Bartomeus et al., 2020; Cejas et al., 2018, 2020; Seabra et al., 2019). One of the reasons for this discrepancy could be the scale of commercial bumblebee operations being conducted in the focal study regions. For example, in the Iberian Peninsula, agricultural practices are both larger in scale, that is 30 000 hectares of greenhouses in Almeria, Spain (Cejas et al., 2018), and use a higher number of commercial bumblebees (300,000 colonies per year in Spain; Cejas et al., 2020, compared to Sweden with ~ 4500 colonies per year; Pedersen et al., 2020). Our study design included a mixture of both small‐ and large‐scale agricultural practices and thus a varied number of commercial bumblebee colonies used per year (Table 1). Although our limited sample size of wild‐caught bumblebees may have affected our capacity to detect introgression, other studies used considerably more wild individuals and did not detect introgression (Hart et al., 2021: ~300 wild‐caught bees; Suni et al., 2017: ~480 wild‐caught bees). The use of microsatellites in these studies may have limited the detection of introgression which suffer from poorer resolution, as discussed by Hart et al. (2021). Data resolution is clearly not a factor in the current study.

We do not know if the commercial *B*. *terrestris* used in this study was *B*. *t*. *terrestris*, and/or *B*. *t*. *dalmatinus*, or hybrids of the two, since both are commonly bred for pollination services in Northern Europe and cannot be distinguished by CO1 barcoding. According to the supplier of commercial bumblebees used by plant growers in this study, bumblebees have a mixed European origin including different subspecies (Pedersen et al., 2020). However, we did not find any significant genetic divergence within our CB samples, indicating that distinct subspecies were either absent or highly admixed, likely because of interbreeding in the breeding facility. Historical or ongoing admixture among different subspecies might also partially explain why we detected more than twice the number of lineages in the CB group compared to the WB group (Figure 2c). If the commercial bumblebees are *B*. *t*. *dalmatinus*, it may be that they are adapted to warmer environments, such that queens and possibly hybrid offspring, have poorer winter survival outside of greenhouses, as this subspecies originated from, and is presumably adapted to, a Mediterranean climate. However, the low level of genetic differentiation between the commercial and wild *B*. *terrestris* group in our study (*F*_ST_ = 0.034, see below) may on the other hand suggest that the commercial and wild bumblebees are of the same subspecies, which would be *B*. *t*. *terrestris*, the most common wild subspecies in Sweden (Rasmont et al., 2008). Unfortunately, we cannot be sure which subspecies are present in the CB group used in this study.

We observed that two of the wild‐caught *B*. *terrestris* (from the experimental sites V2 and T6 sites) were genetically assigned to the commercial *B*. *terrestris* group. These two individuals were most likely foragers from commercial colonies, since foraging distances for *B*. *terrestris* at the distance they were caught (~700 m) are not exceptional (Wolf & Moritz, 2008). Importantly, these individuals do not constitute evidence of establishment of local breeding commercial bumblebee populations. Similarly, Seabra et al. (2019) and Suni et al. (2017) made observations of seven commercial *B*. *terrestris* and eight commercial *B*. *impatiens*, respectively, foraging outside of the greenhouses—escapees. Interestingly, we also assigned two bumblebees collected from commercial hives to the wild group, which may be due to resource theft or drifting behaviour. Similar observations of drifting or resource theft by workers were made by Suni et al. (2017) in commercial colonies of *B*. *impatiens*.

### 4.2 Genetic diversity and divergence between commercial and wild bumblebees

Both groups showed moderate genetic diversity. However, nucleotide diversity (*π)* was higher in wild bumblebees, while the number of segregating sites (*θw)* was higher in commercial bumblebees. The discrepancy between the pattern for these two diversity statistics was supported by significantly positive Tajima's D values in wild bumblebees, which could be indicative of a sudden population contraction. Numerous studies document population contractions and wild bee declines in both abundance and diversity due to anthropogenic change (Goulson et al., 2008; Goulson & Hughes, 2015; Kerr et al., 2012; Kosior et al., 2007; Marshman et al., 2019). However, in our study area of Southern Sweden, the relative abundance of *B*. *terrestris* is known to have increased from 21% to 79% between 1871 and 2015 (Herbertsson et al., 2021; see also similar increase for *Bombus terrestris lusitanicus* in the Iberian Peninsula, Ornosa et al., 2017).

On the other hand, positive Tajima's D values can be indicative of balancing selection, where high heterozygosity is maintained via natural selection, for example as observed in genes involved in the immune response (Ellis et al., 2012). Thus, positive Tajima's D values may represent evidence for balancing selection in wild bumblebees in Southern Sweden. Given the effect of pathogens on wild bee populations globally, and also in commercial colonies (Cameron & Sadd, 2020), studying the role of balancing selection in conferring potential immunity differences between these groups represents interesting future research.

Other studies have found moderately higher genetic diversity (observed heterozygosity and mean allelic richness) in wild compared to commercial bumblebees in the UK (Hart et al., 2021) and New England, USA (Suni et al., 2017), and in mainland Europe (Moreira et al., 2015). Seabra et al. (2019), however, found no difference in genetic diversity (expected heterozygosity) between wild and commercial *B*. *terrestris*. Interestingly, studies that report higher genetic diversity used microsatellite markers, while Silva et al. (2020) and Seabra et al. (2019), who reported similar values of genetic diversity to those detailed here, used RADseq. RADseq and WGS provide a much higher genetic data resolution compared to microsatellite‐based analyses and might provide a more accurate estimate of bumblebee genetic diversity, as discussed by Lozier (2014).

We did not detect significant inbreeding within either the commercial or wild bumblebee groups. However, although inbreeding was low in both groups, there was overall a higher level of global *F*_IS_ among the commercial (*F*_IS_ = 0.044) versus the wild *B*. *terrestris* (*F*_IS_ = −0.009), which may indicate genetic effects of commercial breeding. This result is not consistent with the findings of Moreira et al. (2015) and Seabra et al. (2019) who did not find differences in inbreeding coefficients between wild and commercial *B*. *terrestris*. The level of genetic differentiation (*F*_ST_) evidenced between commercial and wild bumblebees varies in previous studies; Suni et al. (2017) found a high and significant mean *F*_ST_ value of 0.07 between commercial and wild *B*. *impatiens*, while the significant differentiation between commercial and wild *B*. *terrestris* populations ranged from 0.041 to 0.046 in Seabra et al. (2019) and between 0.04 to 0.18 in Kraus et al. (2011). Similar to the *F*_ST_ values reported here (0.034), using microsatellites Hart et al. (2021) reported an *F*_ST_ value of 0.03 between commercial and wild *B*. *terrestris*, suggesting that estimates of *F*_ST_ measured from microsatellites and SNPs are comparable (Lemopoulos et al., 2019).

### 4.3 Identification of a highly divergent region on chromosome 11

We found moderate to low global genetic differentiation between commercial and wild *B*. *terrestris* (median *F*_ST_ = 0.034), but discovered a notable region of high pairwise *F*_ST_ at the tail‐end of chromosome 11 (median *F*_ST_ = 0.04) (Figure 3a). This high peak of *F*_ST_, together with evidence of an excess of extended homozygous haplotypes (Figure 4b) and reduced genetic diversity within this region in the commercial *B*. *terrestris* group, are indicative of differential selection processes operating in wild and commercial *B*. *terrestris*.

We observed evidence for reduced recombination in this region in the form of elevated LD (Figure 4e) and shifts in localized heterogeneity (Figure 4d). Based on a linkage map of *B*. *terrestris* (Stolle et al., 2011), chromosome 11 did not show any evidence of having unusual recombination rates and also showed high homology to *A*. *mellifera*. *B*. *terrestris* is reported to have higher than average genome‐wide recombination rates compared to other insects (Stolle et al., 2011; Wilfert et al., 2007), which lends further support to the hypothesis that chromosome 11 may be under selection in commercial bees. While we did not observe evidence for large structural variants encompassing the entire candidate region on chromosome 11, we did see an increase in the number of structural variants (SVs). However, to determine the role of these SVs and to assess if they are being maintained via linkage, long‐read sequencing would be required as opposed to the short‐read data we analyse in our study. Additionally, we observed an increase in gene density in the diverged region of chromosome 11 (Figure S11), which can also cause signals of chromosomal deviations. Future studies should aim to fully characterize this region and the underlying processes maintaining the differentiation between wild and commercial bees.

We identified 177 genes in the candidate region on chromosome 11. However, since none of the GO terms were significant after multiple‐test correction, we cannot be certain about which genes, or potential pathways may be under selection. We will therefore only briefly discuss the potential relevance of some genes which could be of functional importance in driving differences between wild and commercial bumblebees. Two of the seven genes with the ‘calcium ion binding’ GO term were identified as Troponin C (TnC) and are associated with regulating flight muscle contraction in insects (Bullard & Pastore, 2011; Cao & Jin, 2020; Kržič et al., 2010; Rusu et al., 2017). Two additional genes (sarcomeric α‐actinin and sarcoplasmic calcium‐binding protein 1) were also identified. Sarcomeric α‐actinin acts as structural support in the Z‐line in the flight muscles of insects (Cao & Jin, 2020; Kržič et al., 2010), while the sarcoplasmic calcium‐binding protein 1 has several functions commonly found in muscle and neuronal tissues exclusively in invertebrates (Hermann & Cox, 1995; Pauls et al., 1993). Arguably, recent selection on commercial *B*. *terrestris* workers has taken place under the laboratory conditions within which they are bred. Under such conditions, flight is highly constrained, suggesting that the observed selection on flight muscle genes may actually be for decreased use of the flight muscles.

Moreover, two out of the four genes associated with ‘chitin binding protein’ GO terms were identified as mucin‐5AC and chitinase domain‐containing protein 1 (CHID1). Mucin‐5AC is a glycoprotein (gel‐forming) that is a major component in the mucus lining in both the respiratory tract and stomach in humans (Cornick et al., 2015; Lang et al., 2016), but is also present in the insect gut lining (Ramsey et al., 2017). The insect midgut epithelium is lined by the peritrophic membrane (PM), which primarily consists of chitin and glycoproteins (Wang & Granado, 1997). This acts as a protective barrier against ingested pathogens and aids food digestion (Kuraishi et al., 2011). CHID1 was the second gene found in the candidate region that is involved in chitin binding and has been suggested to play a role in the innate immune response against chitin‐binding pathogens (Ab et al., 2020). *Nosema bombi* and *Crithidia bombi*, two of the most common gut pathogens that infect bumblebees (Geslin et al., 2017), infect via digestion of spore‐infected food or via contaminated bee faeces and develop in the midgut epithelial lining (Paris et al., 2018; Pham & Schneider, 2008). It is possible that the eusocial behaviour of bumblebees selects for increased gut pathogen immunity since it creates a favourable environment for pathogen spread to genetically similar individuals (Simone‐Finstrom, 2017). However, further research is needed to better understand the function of genes that may be selected for in commercial versus wild bumblebees.

## 5 CONCLUSIONS

Human‐mediated introduction of non‐native commercial bumblebees for pollination services is known to have negative ecological effects on some local pollinator fauna and plant–pollinator relationships (Aizen et al., 2019; Evans, 2017; Hingston & McQuillan, 1998; Morales et al., 2013). However, the evolutionary outcome of this remains uncertain. To our knowledge, this is the first study to use whole‐genome sequencing data for the purpose of detecting introgression between wild and commercial *B*. *terrestris*, which allows for higher genetic data resolution compared to reduced marker techniques. We found individuals from the commercial hives foraging up to 700 m from the colony where we collected wild bumblebees, suggesting that hybridization is a possibility if queens and drones also escape into wild environments. The lack of recent introgression in our study suggests that the historical and current extensive use of commercial *B*. *terrestris* in Southern Sweden is yet to affect evolutionary processes within local wild populations of *B*. *terrestris*. However, given our relatively low sample size, we cannot exclude the possibility of introgression between wild and commercial *B*. *terrestris* since commercial individuals were identified among wild bumblebees in the natural environment. Since different subspecies of *B*. *terrestris* differ in traits related to phenology, foraging efficiency, colony size and parasite resistance (Rasmont et al., 2008), divergent selection processes can act on wild and commercial *B*. *terrestris*. Additionally, the discovery of a highly divergent region on chromosome 11 in commercial *B*. *terrestris*, with genes involved in cellular processes associated with flight muscle contraction and immune response, may be indicative of selection differences between wild and commercial bumblebees. Therefore, it is possible that future introgression could result in hybrids with a competitive advantage over wild individuals or lead to maladapted wild populations. This calls for continued investigation into the interactions between wild and commercial bumblebees, especially under unpredictable climate change scenarios.

## 6 SUPPLEMENTARY INFORMATION

The supplementary material contains the protocol for DNA extraction, protocol for COI barcoding, median nucleotide diversity and *F*_ST_ per chromosome, SVs output results from smoove and breakdancer, PCA figure for the CB and WB group, figure of the cross validation (CV) error values from ADMIXTURE analysis *K* = 1–10, CV error values across 10 separate runs with *K* = 1–5, figure of the admixture proportions and standard error from bootstrapped ADMIXTURE runs, figure of the ancestry proportions for each individual sample grouped by the CB and WB groups to the ADMIXTURE run of *K* = 3, figure of the dendrogram visualizing genotypes for the outlier region (1,450,000–17,000,000 kb) on chromosome 11, LD heatmaps for the CB and WB group separately, figure of selection scans performed on chromosome 10 and figure of the density of genes along chromosome 11 for the CB group. The supplementary excel document contains Tables S1–S10.

## CONFLICT OF INTEREST

None declared.

## Supporting information

Supplementary Material

Table S1‐S10

## Data Availability

Whole‐genome sequencing data are available at the European Nucleotide Archive (ENA) under the Study Accession PRJEB49221.
