# Supplementary material for: Genomic divergence and a lack of recent introgression between commercial and wild bumblebees (Bombus terrestris)
Source: Evol Appl. 2022 Mar 14;15(3):365–82. doi: 10.1111/eva.13346 (PMC8965379; doi:10.1111/eva.13346)
Supplement: Supplementary file 1 — Supplementary Material [file EVA-15-365-s002.pdf]

SUPPLEMENTARY MATERIAL

Genomic divergence and a lack of recent introgression between commercial and wild bumblebees (*Bombus terrestris*)

Running Title: Genomics of commercial and wild bumblebees

Cecilia Kardum Hjort[1], Josephine R. Paris[2], Peter Olsson[3], Lina Herbertsson[1], Joachim R. de Miranda[4], Rachael Y. Dudaniec[5*], Henrik G. Smith[1,3*]

*Joint senior authors

Contact Information:

[1] Department of Biology, Lund University, Lund, SE-223 62, Sweden

[2] Biosciences, College of Life and Environmental Science, University of Exeter, Exeter, EX4 4QD, United Kingdom

[3] Centre for Environmental and Climate Research, Lund University, SE-223 62 Lund, Sweden

[4] Department of Ecology, Swedish University of Agricultural Sciences, Box 7044, SE-750 07 Uppsala, Sweden

[5] Department of Biological Sciences, Macquarie University, Sydney, 2109 NSW, Australia

Corresponding Author

Cecilia Kardum Hjort: Department of Biology, Lund University, Lund, SE-223 62, Sweden.

Email: Cecilia.hjort@biol.lu.se

Supplementary Text

Text S1: Purification of DNA from *B. terrestris* for whole genome sequencing using a modified version of QIAGEN Supplementary Protocol:

Purification of total DNA was performed from *B. terrestris* using a DNeasy Blood & Tissue Kit (Qiagen). The head and two legs from each individual were removed using a pair of tweezers. Ethanol was allowed to evaporate from each sample prior to extraction by letting the tissue try for 10 minutes on tissue paper. The tissue was then placed in 2 ml Cryo-tubes together with 500 µl of Buffer SET and a 5 mm magnetic steel bead and further homogenized in the TissueLyser at 20 Hz for 2 min. The fragments were then spun down briefly in order to remove the steel beads (with a magnet) without losing any fragments. Then, the fragments were spun down to a pellet at 20 000 x g for 3 min. The Buffer SET was removed (without disrupting the pellet) and discarded using a pipette.

The pellet together with Buffer ATL and proteinase K was incubated in a heating cupboard at 56°C overnight (approx. 10-18 hours). On day 2, 4 µl RNase A (100 mg/ml; 7000 units/ml) was added to each sample and pulse vortexed 15 times, spun down briefly and incubated for 30 minutes at 37°C. EB buffer was added to a 2 ml cryo-tube and put in the heating cupboard at 60°C. Then 200 µl Buffer AL and 200 µl 99.5 % EtOH was added to each sample (note: these had been mixed together before hand) and pulse vortex 10 times, then centrifuge at 20 000 x g for 3 min.

The DNA was eluted in two steps: 50 µl of heated EB Buffer was added directly onto the membrane to the Mini spin column without touching it. Then incubated at room temperature for 3 minutes, then centrifuged at 600x g for 1 minute to elute the DNA. This step was repeated once more.

Text S2: **COI Barcoding**

The COI mitochondrial gene was amplified in all samples using the universal primer-pair: HybLCO 5' TAA TAC GAC TCA CTA TAG GGG GTC AAC AAA TCA TAA AGA TAT TGG 3' and HybHCO 5' ATT AAC CCT CAC TAA AGG GT AAA CTT CAG GGT GAC CAA AAA ATC A 3'. The PCR cycling conditions were as follows: 95°C for 5 min, 40 cycles of 94°C for 30 sec, 50°C for 30 sec and 72°C for 1 min 30 sec, with a final extension period of 72°C for 10 min. The PCR product was purified using 0.9 µl of ExoI (ThermoFisher Scientific) accordingly to Wahlberg & Wheat (2008) and submitted to Macrogen Europe (https://dna.macrogen-europe.com/) for Sanger sequencing. The generated COI sequences were compared with both complete and partial mitochondrial genome sequences from *B. terrestris* in GenBank using Nucleotide BLAST to confirm that all samples were *B. terrestris*. A 100 % match rate was used as a threshold to identify each sample.

Supplementary Figures

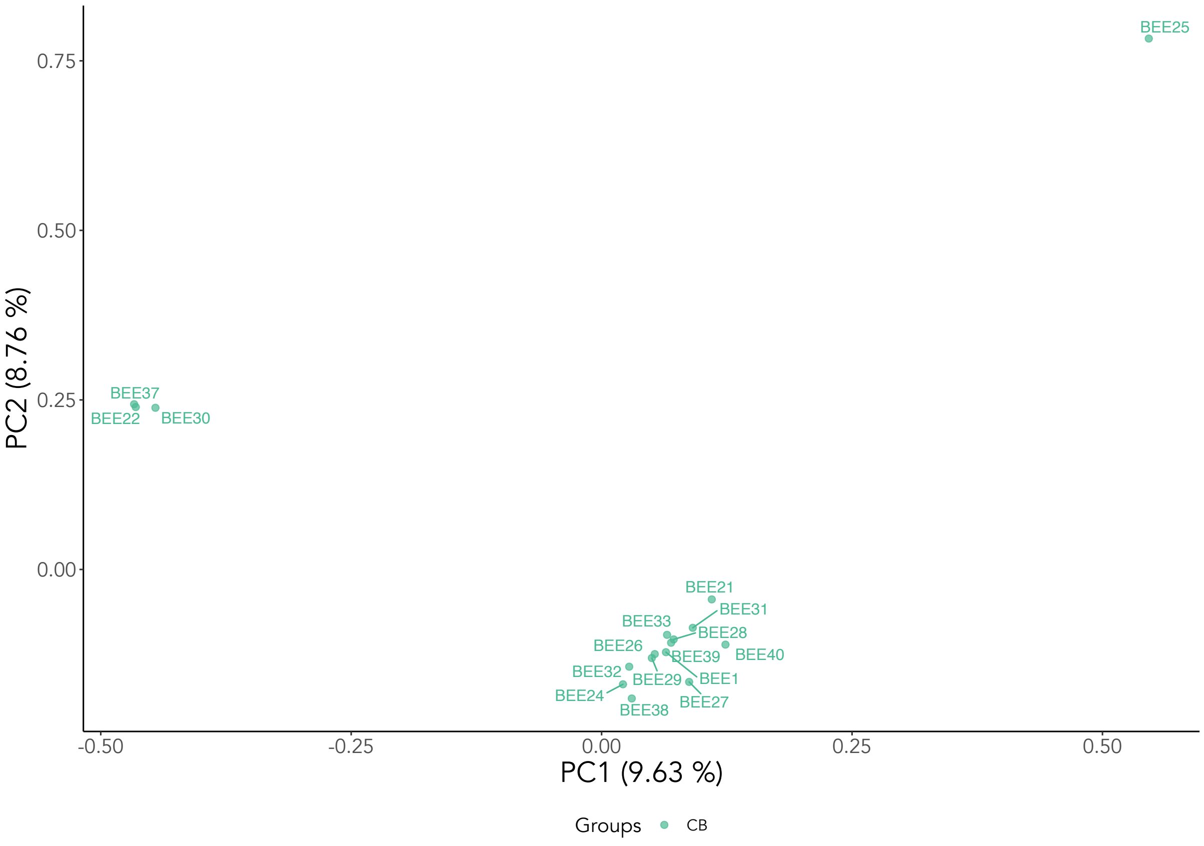

Figure S1. PCA plot of the commercial group (CB) consisting of 17 individuals, showing the genetic relationship among individuals within the same cluster. No clear substructure is visible within the CB group. Samples 22, 30 and 37 are the believed wild “drifters”, i.e., bees that belong to the wild bumblebee genetic cluster that were sampled within non-natal commercial hives. Sample 25 is seen as an outlier.

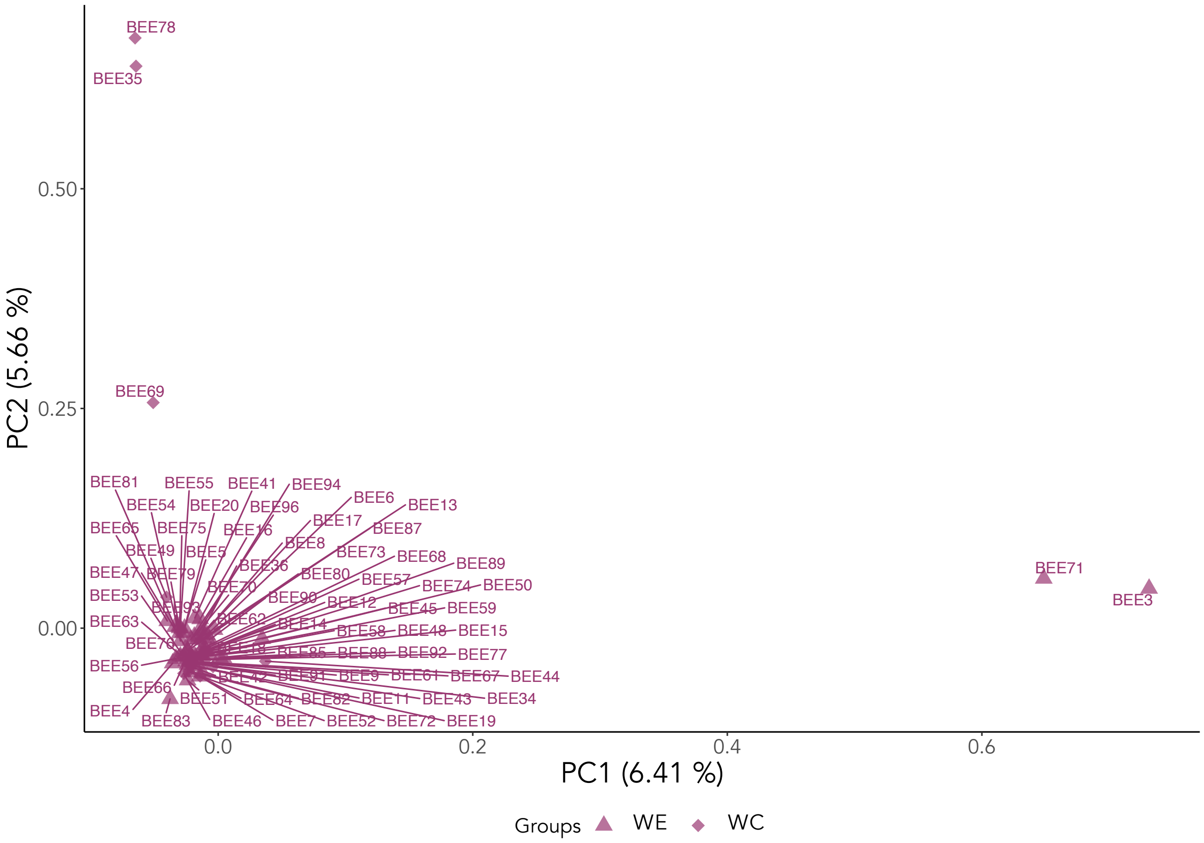

Figure S2. PCA plot of the WB group consisting of 72 individuals, showing the genetic relationship among individuals within the same cluster. No clear substructure is defined. Arguably, sample 78 and 35 separates from the main cluster. These two samples are collected from the same site (2nd degree siblings, kinship coefficient of 0.100). Samples 3 and 71 are the believed commercial “escapees" and are separate from the main cluster. WE= wild exposed, WC= wild control.

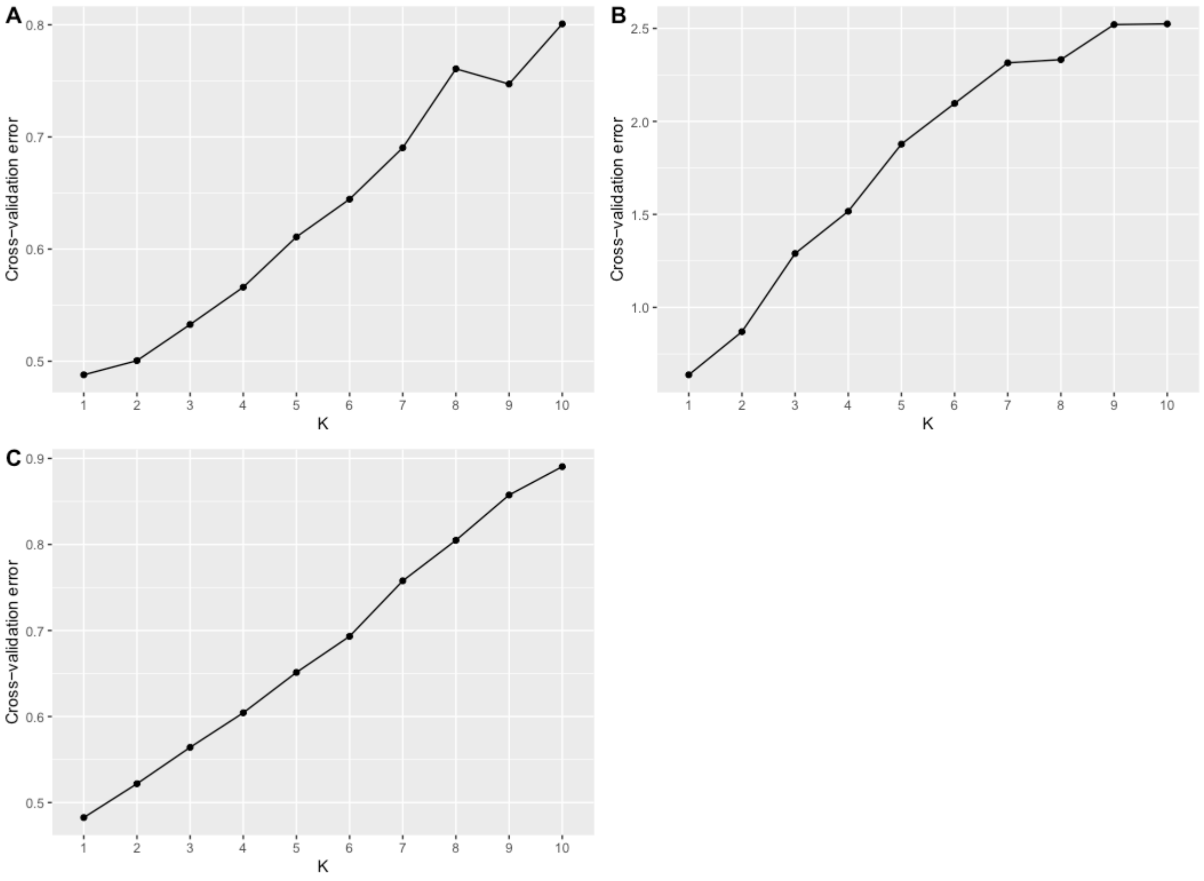

Figure S3. Cross validation (CV) error values from ADMIXTURE analysis with K values of 1-10 shown with a 10-fold CV. A low CV error value indicates a well-supported K value compared to the other K values. A) whole dataset B) subset WB C) subset CB

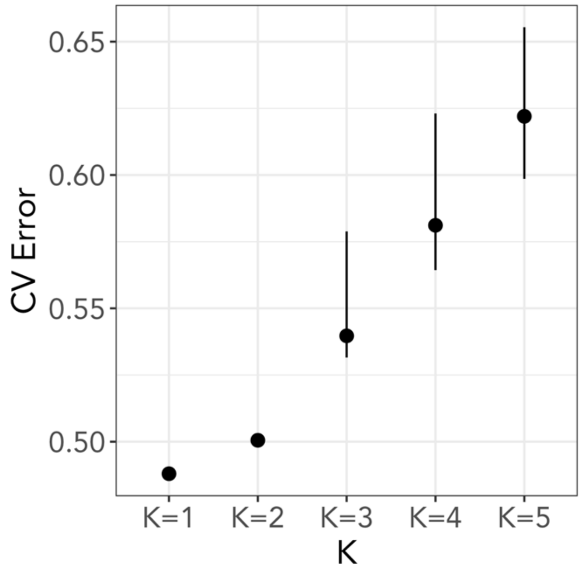

Figure S4. Variance in CV error between 10 different ADMIXTURE runs. K=1 and K=2 showed minor fluctuations in the CV error compared to higher values of K.

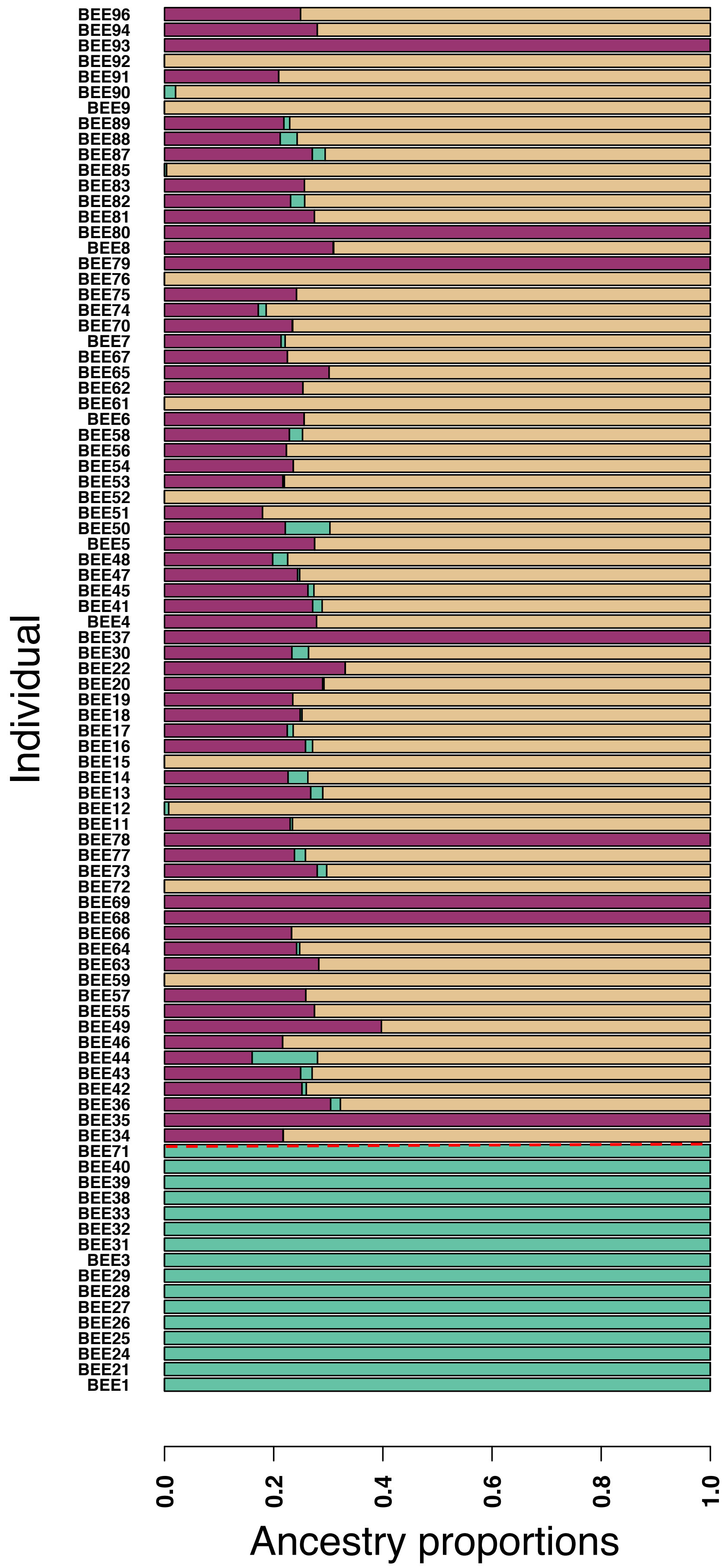

Figure S5. Ancestry proportions for each individual sample (n=89) grouped by the CB and WB groups to the ADMIXTURE run of K=3. Cluster 1 (green, CB) consists of commercial bumblebees. The WB group (purple and beige) forms two clusters, however K=3 has low support (see Figure S5) and therefore should not be used. No sign of admixed individuals between the WB and CB group is evident. The red dashed line separates the two groups.

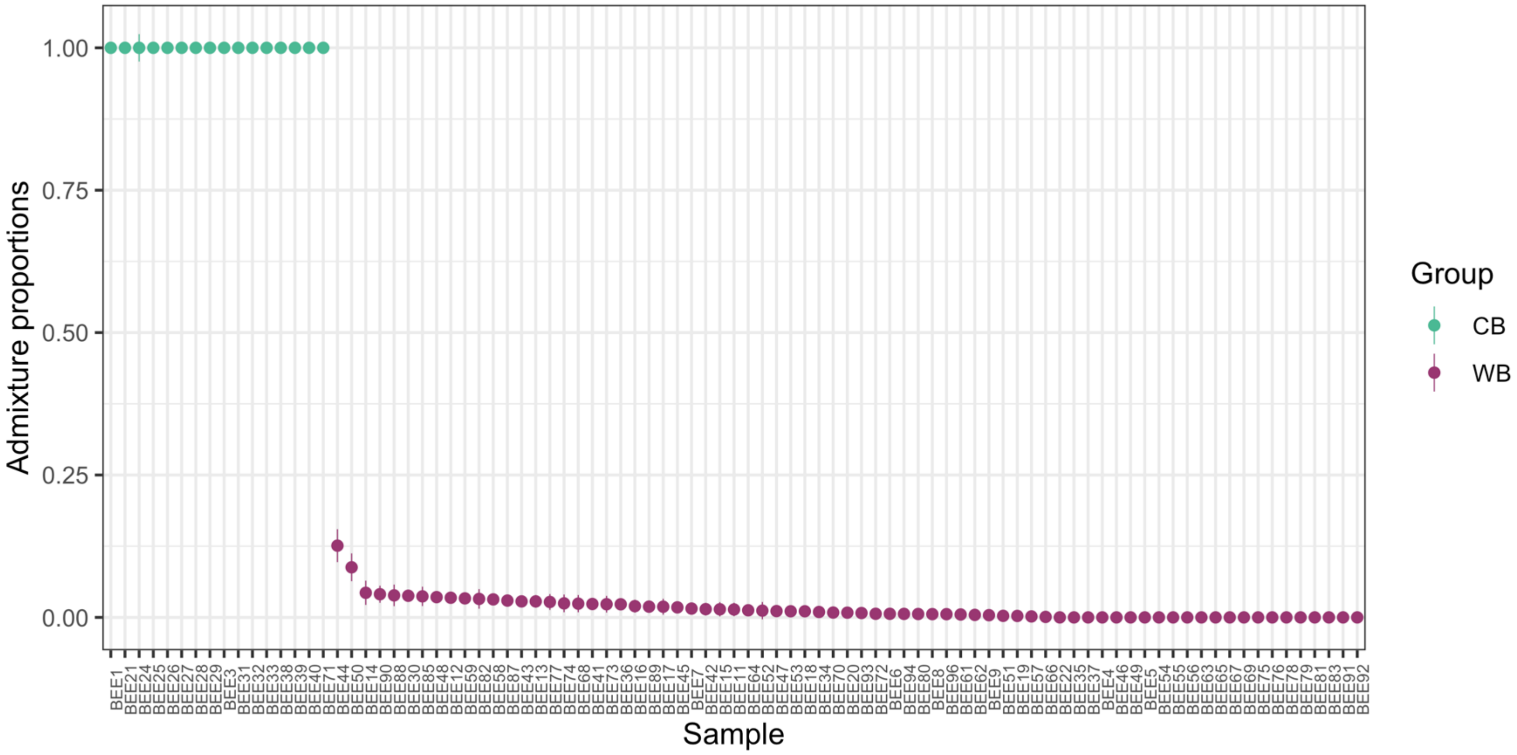

Figure S6. Admixture proportions and standard error from ADMIXTURE. Mean is average taken across 10 runs. Standard error is calculated from 1000 bootstraps of the data. Points are coloured as per Figure 2.

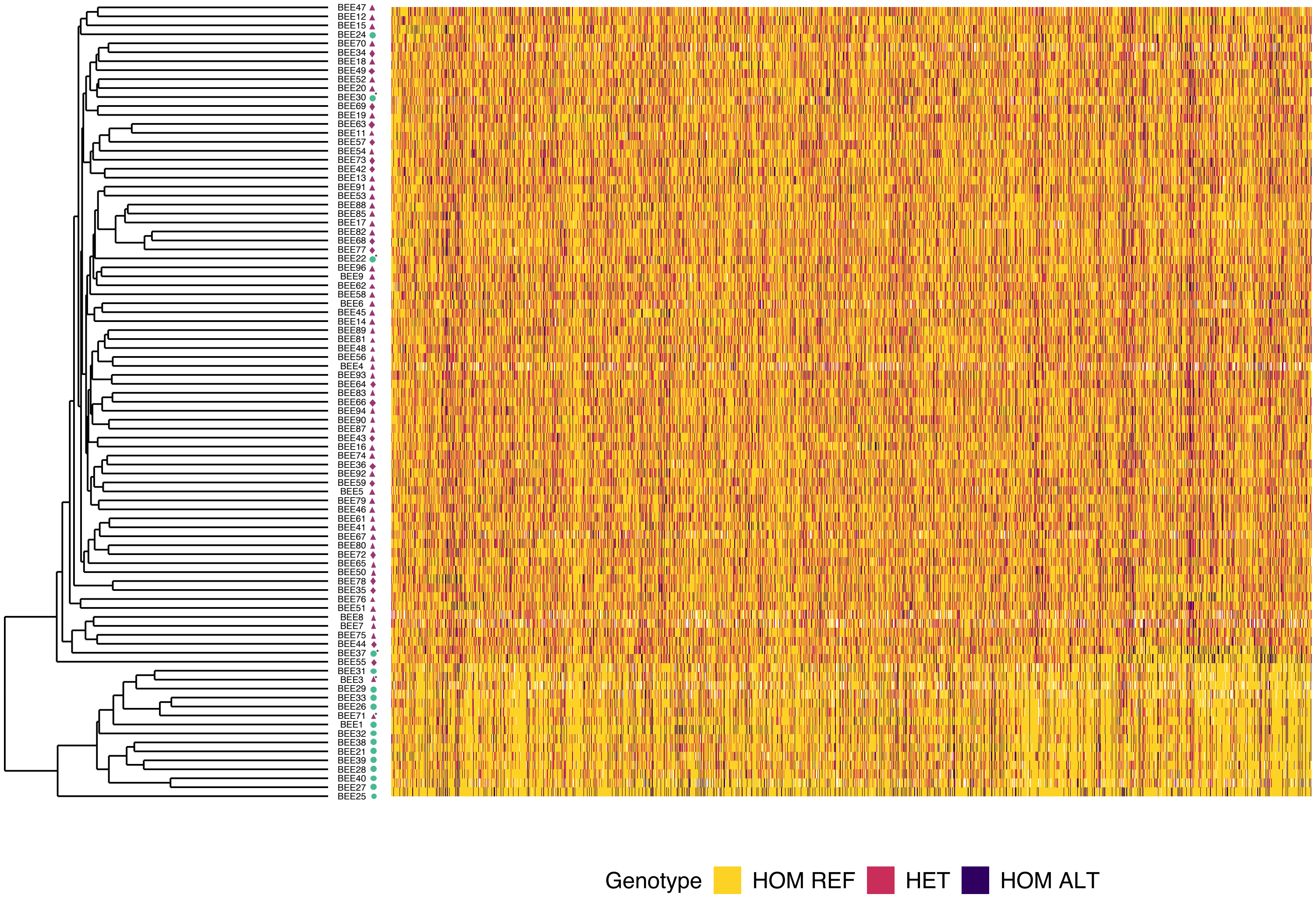

Figure S7. Dendrogram visualising genotypes for the outlier region (1450000-17000000 kb) on chromosome 11 (dataset with N= 89 individuals). Samples belonging to the CB group are shown as green circles, samples belonging to the WE group as purple triangles and samples belonging to WC as purple diamonds. '*' Indicate the samples that were later defined as "drifters" (22, 30 and 37) and "escapees" (samples 3 and 71).

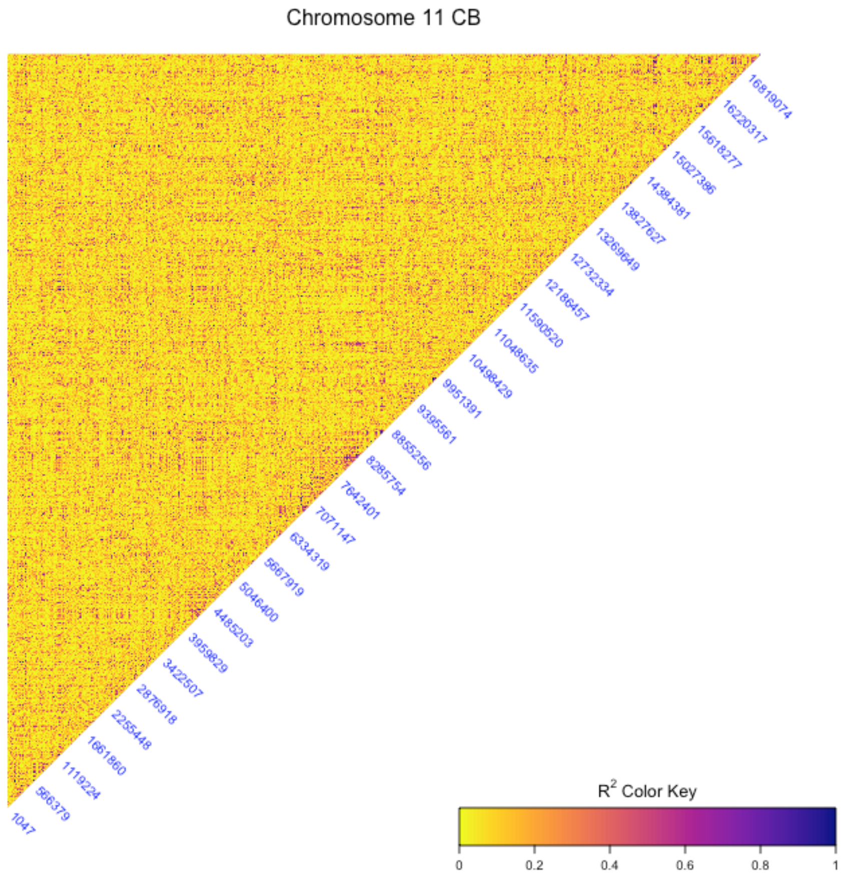

Figure S8. Pairwise linkage disequilibrium (LD) heatmap for the CB group, calculated using squared r. Chromosome 11.

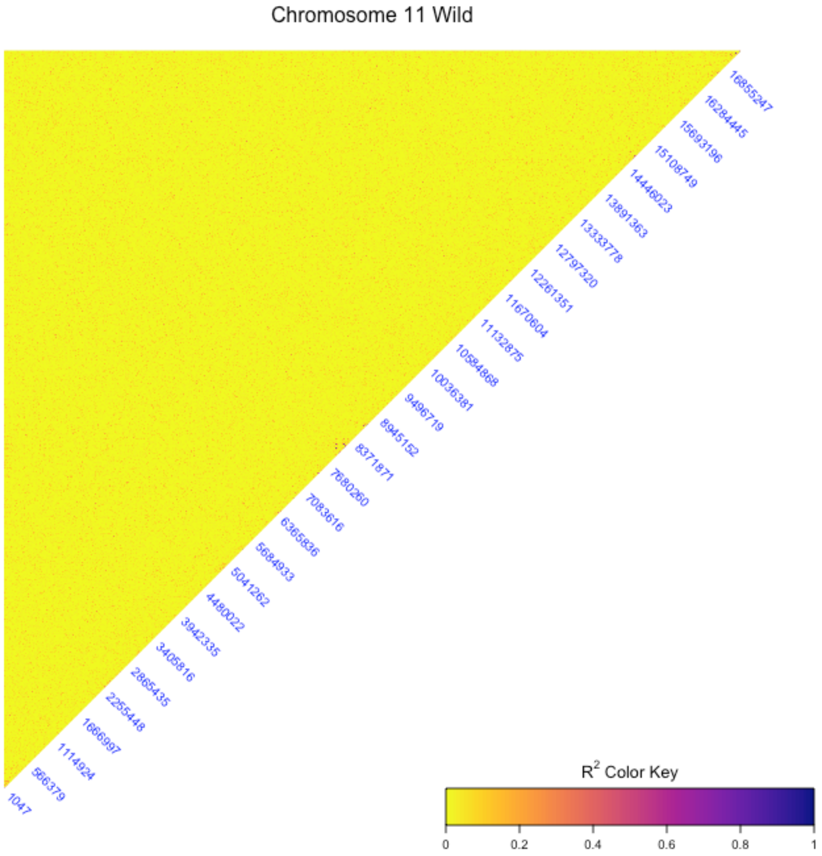

Figure S9. Pairwise linkage disequilibrium (LD) heatmap for the WB group, calculated using squared r. Chromosome 11.

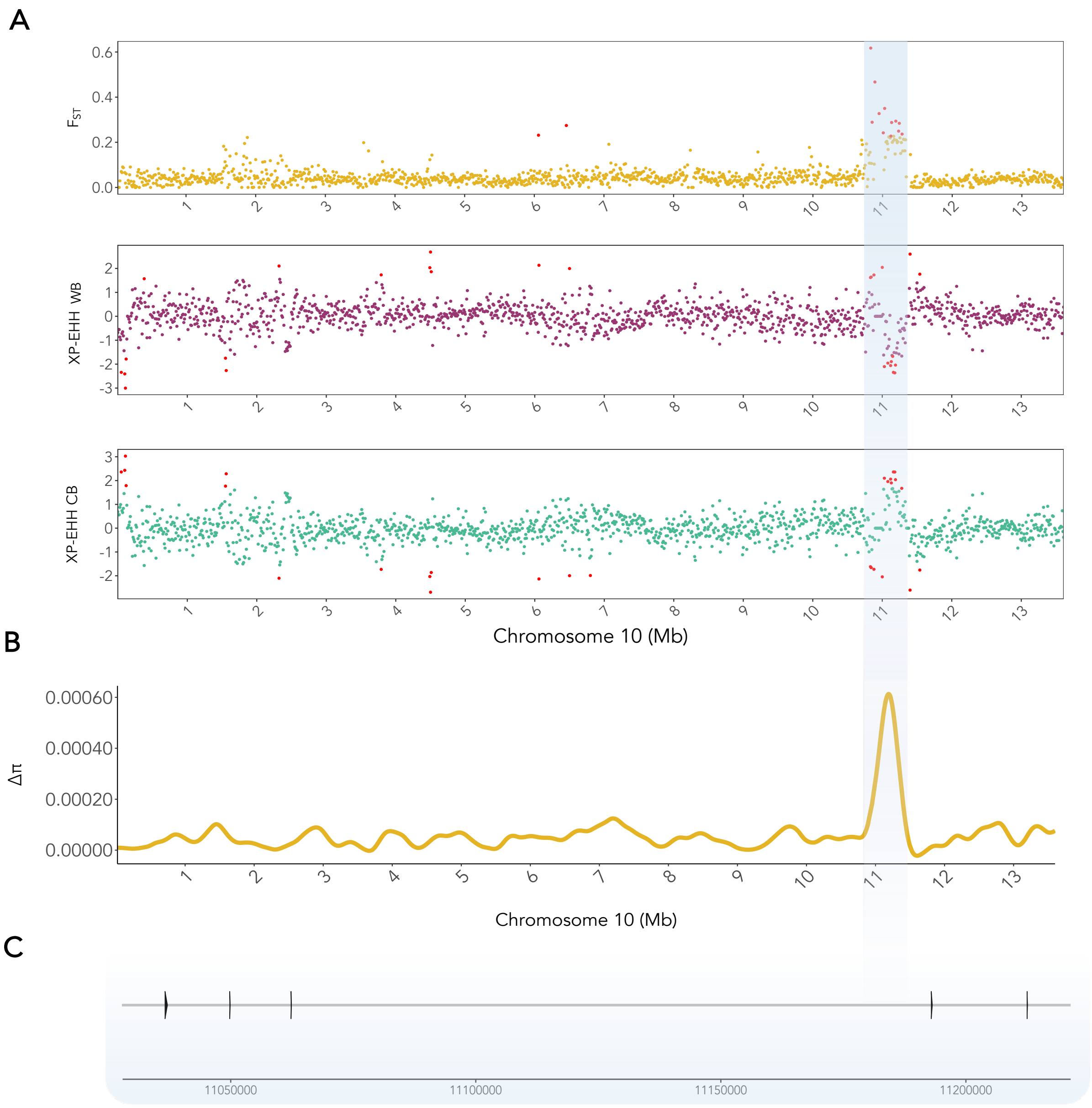

Figure S10. A) Pairwise F_{st} for chromosome 10 (upper panel, yellow), standard mean XP-EHH score for chromosome 10 for the WB group (middle purple panel) and the CB group (lower green panel), where the light blue shading highlights the windows containing significant outlier SNPs (in red). The red SNPs indicate the upper 99 % and lower 1 % confidence intervals B) Delta nucleotide diversity along chromosome 10. The light blue shading highlights the difference in nucleotide diversity between the two groups at chromosome 10 C) annotated genes (n=5) for the significant outlier SNPs on chromosome 10.

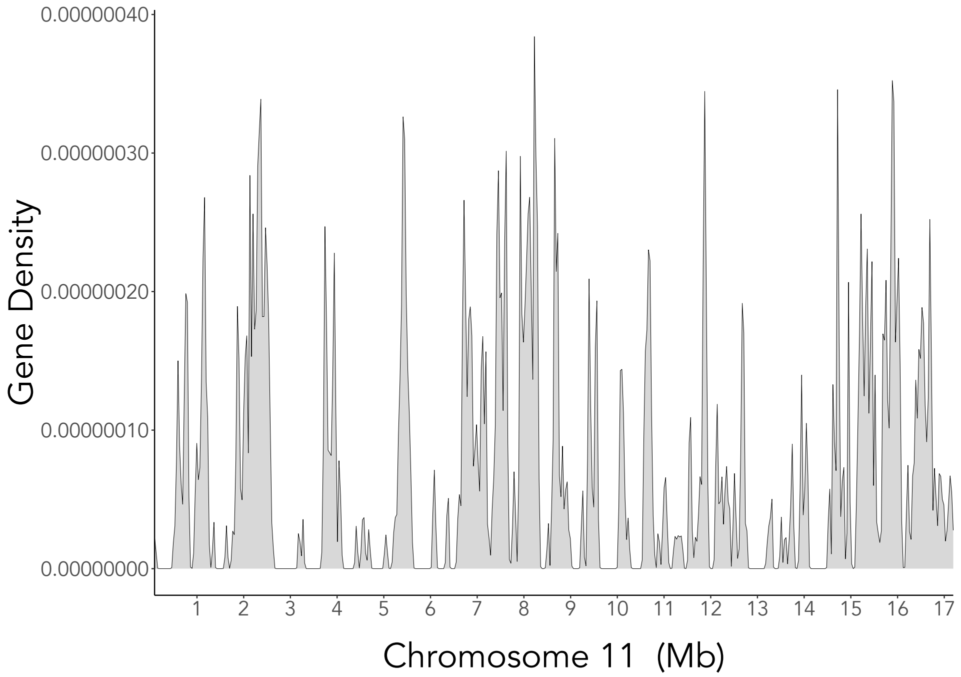

Figure S11. Density of genes along chromosome 11 for the CB group. A slight increase in gene density is visible for the candidate region (14.5-17.07 Mb).
